# Pure cerebellar ataxia due to bi‐allelic PRDX3 variants including recurring p.Asp202Asn

**DOI:** 10.1002/acn3.51874

**Published:** 2023-08-08

**Authors:** Stephanie Efthymiou, Luiz E. Novis, Georgios Koutsis, Chrysoula Koniari, Reza Maroofian, Valentina Turchetti, Georgios Velonakis, Luiz F. Vasconcellos, Salmo Raskin, Varunvenkat M. Srinivasan, Alistair T. Pagnamenta, Yaramanchanahalli B. Arun, Uddhava V. Kinhal, Vykuntaraju K. Gowda, Helio A. G. Teive, Henry Houlden

**Affiliations:** ^1^ Department of Neuromuscular Disorders UCL Queen Square Institute of Neurology London WC1N 3BG UK; ^2^ Neurological Diseases Group, Postgraduate Program of Internal Medicine Hospital de Clínicas, Federal University of Paraná Curitiba Paraná Brazil; ^3^ Neurogenetics Unit, 1st Department of Neurology Eginition Hospital, National and Kapodistrian University of Athens Athens Greece; ^4^ 2nd Department of Radiology Medical School, Attikon Hospital, National and Kapodistrian University of Athens Athens Greece; ^5^ Institute of Neurology, Federal University of Rio de Janeiro Rio de Janeiro Brazil; ^6^ Genetika Laboratoty Curitiba Paraná Brazil; ^7^ Department of Pediatric Neurology Indira Gandhi Institute of Child Health Bangalore India; ^8^ NIHR Biomedical Research Centre, Wellcome Centre for Human Genetics University of Oxford Oxford UK

## Abstract

Bi‐allelic variants in peroxiredoxin 3 (*PRDX3*) have only recently been associated with autosomal recessive spinocerebellar ataxia characterized by early onset slowly progressive cerebellar ataxia, variably associated with hyperkinetic and hypokinetic features, accompanied by cerebellar atrophy and occasional olivary and brainstem involvement. Herein, we describe a further simplex case carrying a reported *PRDX3* variant as well as two additional cases with novel variants. We report the first Brazilian patient with SCAR32, replicating the pathogenic status of a known variant. All presented cases from the Brazilian and Indian populations expand the phenotypic spectrum of the disease by displaying prominent neuroradiological findings. SCAR32, although rare, should be included in the differential diagnosis of sporadic or recessive childhood and adolescent‐onset pure and complex cerebellar ataxia.

## Introduction

SCAR32 is a novel autosomal recessive spinocerebellar ataxia caused by bi‐allelic variants in *PRDX3* (OMIM 604769), the gene encoding for peroxiredoxin 3.^1^ PRDX3 is a mitochondrial antioxidant enzyme that catalyzes the reduction of hydrogen peroxide and is instrumental to reactive oxygen species homeostasis. From two previously reported knockout mouse lines, we know that PRDX3 deficiency can lead to reduced mitochondrial DNA content and ATP production with impaired mitochondrial fusion as well as impaired glucose tolerance and insulin resistance, important for metabolic homeostasis.^2^, ^3^

Rebelo *et al*.^1^ first described five simplex cases with early onset (13–23 years) slowly progressive cerebellar ataxia, variably associated with hyperkinetic and hypokinetic features, accompanied by cerebellar atrophy and occasional olivary and brainstem involvement. All cases carried bi‐allelic loss‐of‐function variants in *PRDX3* that were found to impair the assembly of the PRDX3 protein complex, leading to the complete absence of PRDX3, impaired cellular response to oxidative stress and mitochondrial dysfunction.

Subsequently, a novel homozygous *PRDX3* variant was reported in a patient with infantile‐onset severe cerebellar ataxia and peripheral neuropathy, expanding the disease's clinical spectrum.^4^, ^5^ Martínez‐Rubio *et al*.^5^ showed with biochemical analysis of the *PRDX3* mutation p.Asp163Glu results in an unstable structure tending to form aggregates that trigger unfolded protein responses via mitochondria and endoplasmic reticulum. More recently, two further families carrying novel homozygous *PRDX3* variants were identified, following a screen of >3,500 ataxia exomes.^6^ One case had infantile‐onset cerebellar ataxia, but the other first developed ataxic gait at age 35, indicating that *PRDX3*‐associated disease may have a broader age of onset range than previously thought. In addition, there was a third report that associates a PRDX3 non‐sense variant p.Lys166* with cerebellar ataxia and profound hearing impairment.^7^

Herein, we present a further simplex case carrying a reported homozygous variant in *PRDX3* as well as two additional cases with novel bi‐allelic variants. The variants were identified by screening of exome data from a large cohort of patients with cerebellar ataxia (>1,200 cases) of multi‐ethnic background.

## Methods

This study was approved by the Ethics Committee of University College London Hospital NHS Foundation Trust (UCLH), the Hospital de Clínicas Ethics Committee (Federal University of Paraná) and the Indira Gandhi Institute of Child Health. Written informed consent was obtained from the patients and parents. To investigate the genetic cause of the disease, whole‐exome sequencing of the DNA from patients was performed as previously described.^8^

## Results

### Clinical phenotype

**Figure 1:**
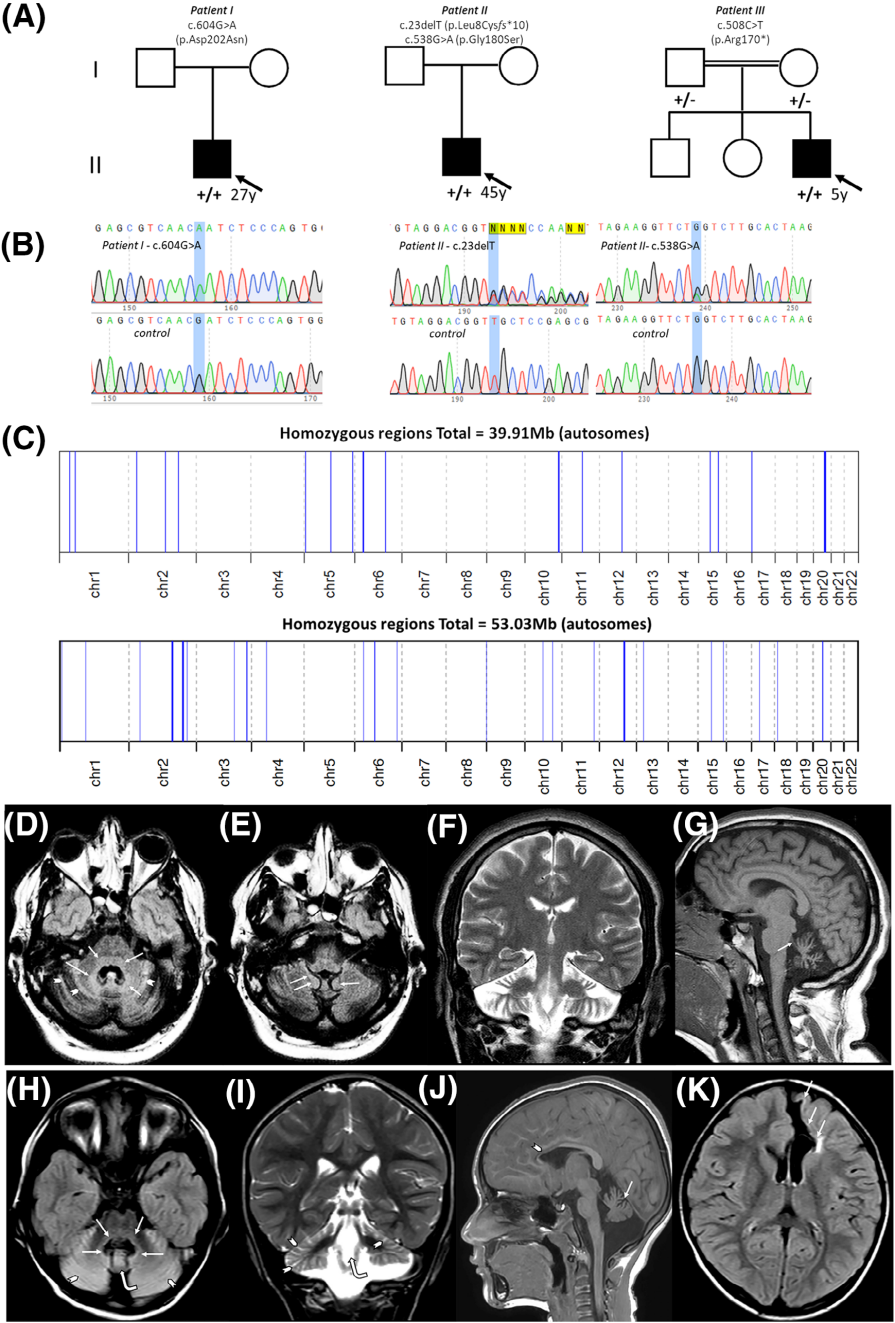
(A) Pedigree information for the simplex Brazilian cases with bi‐allelic *PRDX3* mutation. (B) Electropherograms of the Sanger sequencing of proband I showing homozygous c.604G>A (p.Asp202Asn) mutation as well as proband II showing compound heterozygous c.23delT (p.Leu8Cysfs*10) and c.538G>A (p.Gly180Ser). Variants are annotated as + (present) and − (absent). (C) Overview of the whole regions of homozygosity (ROH) in the exome of non‐consanguineous proband I (top) and consanguineous proband III (below). A 6.45 Mb region of homozygosity surrounds the *PRDX3* variant in the case of proband I. (D‐K) MRI brain imaging from patient I: (D, E) Axial Τ2 Fluid‐attenuated inversion recovery (FLAIR) images reveal cerebellar atrophy. T2 hyperintensity is noticed around the fourth ventricle (arrows), including the posterior pons and the formation reticularis, the medial part of bilateral middle cerebellar peduncles, the dentate nuclei and the anterior cerebellar vermis. Hyperintensity is also seen in cerebellar gyri (arrowheads) as well as in the superior edge of the cerebellar tonsils (arrows). Coronal T2‐weighted image (F) shows prominent cerebellar atrophy compared with the supratentorial structures. Sagittal T1 image (G) reveals vermis atrophy and thinning of the superior cerebellar peduncles (arrow). (H–K) MRI brain imaging from patient III: (H) Axial Τ2 Fluid‐attenuated inversion recovery (FLAIR) image (A) reveals dilatation of the fourth ventricle. T2 hyperintensity is seen around the fourth ventricle (arrows), including the posterior pons and the formation reticularis, the medial part of bilateral middle cerebellar peduncles, the dentate nuclei and the anterior cerebellar vermis (curved arrow). T2 hyperintensity is also seen in the cerebellar gyri (arrowheads). Cerebellar atrophy is more evident in the coronal T2‐weighted image (I), which shows an enlargement of the fourth ventricle (curved arrow) gyri T2‐hyperintensity and sulci dilatation (arrowheads). Sagittal T1 image (J) reveals cerebellar vermis atrophy (arrow). Thinning of the anterior part of the body and the genu of the corpus callosum (arrowhead) is attributed to encephaloclastic porencephaly shown on the axial FLAIR image (K) Parenchymal cavity extends to the ependymal of the frontal horn of the left lateral ventricle and it is lined by gliotic white matter (arrows).

Patient I (p.Asp202Asn) is a 27‐year‐old male from a non‐consanguineous Brazilian family with pure cerebellar syndrome. He reported insidious onset of gait ataxia and dysarthria around the age of 18 years, with limb ataxia developing shortly thereafter. The disease course was slowly progressive, and the patient can still walk independently 10 years following symptom onset. He reported no symptoms of dysautonomia. There is no family history of ataxia (Fig. 1A). Both parents are Brazilian, originally from different cities in the Paraíba state, located in the northeast region, with no evidence of consanguinity. The patient has no other siblings. On neurological examination, he exhibited moderate gait ataxia, cerebellar dysarthria, saccadic pursuit, multi‐directional nystagmus, four limbs dysmetria, comprising a scale for the assessment and rating of ataxia (SARA) score of 12/40. He also had brisk deep tendon reflexes, but no Babinski sign. There was no evidence of cognitive impairment, extrapyramidal or pyramidal tract involvement, autonomic dysfunction, or peripheral neuropathy. On clinical examination, the patient exhibited micropenis, with no other changes. His testicles were of normal size and consistency, as was the distribution of hair over his body. An endocrinological exam showed that the patient presented with low levels of follicle‐stimulating hormone (FSH) at 1.9 mIU/mL, luteinizing hormone (LH) at 1 mIU/mL, testosterone at 0.2 mg/mL and dihydrotestosterone (DHT) at 0.01 mg/mL. These laboratory findings confirmed the presence of hormonal dysfunction. To address the hormonal deficiency, a treatment regimen involving intramuscular administration of human chorionic gonadotropin (HCG) at a dose of 500 IU once a week for 4 weeks was initiated. Subsequent laboratory evaluations revealed an increase in testosterone levels to 6.42 mg/mL, while DHT levels remained unchanged at 0.01 mg/mL. In 2018, the patient underwent plastic surgery to correct micropenis and hormone measurements was performed during this period. The results showed testosterone levels at 4.15 mg/mL, FSH at 5.32 mIU/mL and LH at 6.04 mIU/mL. A brain MRI scan, performed at the age of 24, showed extensive cerebellar and brainstem hyperintensities. More specifically, there were findings of T2 hyperintensities noticed at the posterior pons and the formation of reticularis, the medial part of bilateral middle cerebellar peduncles, the dentate nuclei and the anterior cerebellar vermis. Vermis atrophy and thinning of the superior cerebellar peduncles were also seen (Fig. 1D–G). Presenting as sporadic ataxia, the patient was extensively investigated for acquired causes of cerebellar ataxia. The extensive biochemical, haematological and immunological screening was unremarkable. Additionally, serum levels of alpha‐fetoprotein, vitamin E, vitamins B1 and B12, copper and ceruloplasmin were normal. Autoantibody testing for celiac disease, other autoimmune cerebellar ataxias (including anti‐GAD) and paraneoplastic cerebellar syndromes were negative. Subsequently, targeted genetic testing for a hereditary cerebellar ataxia was performed. DNA analysis for spinocerebellar ataxia (SCA) 1, 2, 3, 6, 7 and 10 as well as Friedreich's ataxia (FRDA) were negative.

Patient II (p.Leu8Cysfs*10; p.Gly180Ser) is a 46‐year‐old male, with no previous family history and a pure cerebellar ataxia phenotype. He began his symptoms at the age of 35 years old. At first, he reported dizziness and gait ataxia that slowly progressed to limb ataxia. Upon neurological examination, he displayed gait ataxia, mild cerebellar dysarthria, dysmetria and dysdiadochokinesia. He is still able to walk without support and no dysphagia was reported. Bidirectional horizontal nystagmus was also noted. His SARA score was 12/40. There was no pyramidal, extrapyramidal, cognitive, peripheral or cranial nerve involvement. A brain MRI scan performed at the age of 45 years old reported a diffuse cerebellar atrophy with no other abnormalities. A nerve conduction study (NCS) and video‐head‐impulse test were also performed without any remarkable findings. His parents are deceased so segregation studies could not be performed.

Patient III (p.Arg170*) is a 5‐year‐old male child of Indian origin born to consanguineous parents (distant relatives). The birth was normal but soon after, at 6 months of age, he presented with global developmental delay, failure to achieve milestones and slowly progressive imbalance in gait at 3 years of age. He achieved walking at 2 years of age and could walk up and down stairs at 4 years old with handrail support. Upon neurological examination, he presented with axial and appendicular skeleton hypotonia, as well as brisk deep tendon reflexes and extensor plantar on both sides. Cerebellar examination showed gait ataxia and gaze‐evoked nystagmus. The skull and spine examination was normal. Cardiovascular, respiratory and abdomen examination was also unremarkable. Head circumference was 48 cm (*Z*‐score −1.83), weight was 18.3 kg (*Z*‐score −0.01) and height was 99 cm (*Z*‐score −2.37). Serum immunoglobulin, AFP, eye examination and hearing assessment were all normal. A brain MRI scan performed at the age of 5 years old reported T2 hyperintensities at posterior pons, formation reticularis, medial part of bilateral middle cerebellar peduncles, dentate nuclei, anterior cerebellar vermis and cerebellar gyri. Additionally, frontal encephaloclastic porencephaly with thinning of the corpus callosum was identified which could be attributed to a congenital or acquired process. The patients' clinical details, along with data from previously reported cases with bi‐allelic *PDRX3* mutations, are presented for comparative purposes in Table 1.

**Table 1:** Clinical spectrum of all reported patients with *PRDX3*‐associated cerebellar ataxia.

	This study			Rebelo *et al*., 2021					Martinez‐Rubio *et al*., 2022	Rebelo *et al*., 2022		Rafeeq *et al*., 2023
Ethnicity	Brazilian	Brazilian	Indian	Kurdish	Kurdish	German	Indian	French	Moroccan	Kurdish	Turkish	Pakistani
Consanguinity	No	No	Yes	Unknown	Yes	Unknown	Yes	–	Yes	Yes	Yes	Yes
Mutations in *PRDX3*	p.Asp202Asn (homozygous)	p.Leu8Cysfs*10 p.Gly180Ser	p.Arg170* (homozygous)	p.Asp202Asn (homozygous)	p.Asp202Asn (homozygous)	p.Ala114Glyfs*3 (homozygous)	p.Arg170* (homozygous)	p.Ala142Gly p.Ser12fs	p.Asp163Glu (homozygous)	p.Arg15* (homozygous)	p.Gln220* (homozygous)	p.Lys166* (homozygous)
Sex	Male	Male	Male	Male	Male	Female	Male	Male	Male	Female	Male	Female
Age at onset (years)	18	35	0.5 with GDD	22	13	23	21	15	<2	2	35	<2
Age at last exam (years)	27	46	5	35	36	54	31	39	6.5	18	51	15
Gait ataxia	Yes	Yes	Yes	Yes	Yes	Yes	Yes	Yes	Yes	Yes	Yes	Yes
Limb ataxia	Yes	Yes	Yes	Yes	Yes	Yes	Yes	Yes	Yes	Yes	Yes	Yes
Dysarthria	Yes	Yes	Yes	Yes	Yes	Yes	Yes	Yes	Yes	‐	Yes	Yes
Oculomotor signs	Saccadic Pursuit Gaze‐evoked nystagmus	Gaze‐evoked nystagmus	Gaze‐evoked nystagmus	Saccadic pursuit Hypermetric saccades	Saccadic pursuit Gaze‐evoked nystagmus	Saccadic pursuit Hypermetric saccades	Saccadic pursuit Hypermetric saccades	Saccadic pursuit	Saccadic pursuit	Hypometric saccades	Saccadic pursuit Hypermetric saccades	Saccadic pursuit
Myoclonus	–	–	–	Yes	–	–	–	Yes	–	–	–	–
Dystonia	–	–	–	–	–	Yes	–	Yes	–	–	–	–
Postural or action tremor	–	–	–	–	–	Yes	–	Yes	Yes	–	–	–
Parkinsonism	–	–	–	–	–	Hypomimia	–	Bradykinesia	–	–	Hypomimia	–
Pyramidal signs	–	–	–	–	–	–	–	–	–	–	–	–
Sensory signs	–	–	–	–	–	–	–	–	–	–	–	–
Cognitive impairment	–	–	–	–	–	–	–	Learning disability	–	–	–	Learning disability
SARA (at last exam)	12	12	8	14	13.5	21.5	8.5	15	19	7.5	12	10.5
Cerebellar atrophy on MRI	Yes	Yes	Yes	Yes	Yes	Yes	Yes	Yes	Yes	Yes	Yes	Yes
Brainstem atrophy on MRI	–	–	–	–	Yes	–	–	–	–	–	–	–
Other MRI findings	T2 hyperintensities at posterior pons, formation reticularis, medial part of middle cerebellar peduncles, dentate nuclei and anterior cerebellar vermis and cerebellar gyri	–	T2 hyperintensities at posterior pons, formation reticularis, medial part of bilateral middle cerebellar peduncles, dentate nuclei, anterior cerebellar vermis, cerebellar gyri. Frontal encephaloclastic porencephaly with thinning of the corpus callosum		Ηyperintense signals at the level of medullary olives	Mild parietal atrophy Ηyperintense signals at the level of medullary olives	–	–	Cerebellar cortical hyperintense signals	Cerebellar and brainstem hyperintense signals	Cerebellar and brainstem hyperintense signals	–
NCS and EMG	ND	Normal	ND	ND	ND	ND	ND	ND	Sensorimotor neuropathy	ND	ND	ND

SARA, scale for the assessment and rating of ataxia; NCS, nerve conduction studies; EMG, electromyogram; ND, not done.

### Genetic analysis

A homozygous missense variant in *PRDX3* (NM_006793.5): c.604G>A, p.(Asp202Asn) residing within a 6.45 Mb region of homozygosity (Fig. 1C) was identified and confirmed by Sanger Sequencing in proband I. Compound heterozygous missense variants in *PRDX3* (NM_006793.5): c.23delT p.(Leu8Cysfs*10) and c.538G>A, p.(Gly180Ser) were identified and confirmed by Sanger Sequencing in proband II (Fig. 1B). Parents were either not available or deceased at the time for genetic testing.

A homozygous non‐sense variant in *PRDX3* (NM_006793.5): c.508C>T, p.(Arg170*) residing within a 2.22 Mb region of homozygosity was identified in Patient III (Fig. 1C). All variants are predicted to be ‘pathogenic’ according to the ACMG (American College of Medical Genetics) criteria, including the very strong PVS1 criterium for null variants and the strong PM2 criterium for extreme low allele frequency for all variants in population databases (Fig. 1D).

Analysis on the 100k Genomes Project data set performed within a secure workspace called the ‘Research Environment’ identified an additional patient, a white British boy with cerebellar hypoplasia, with the homozygous missense variant in *PRDX3* (NM_006793.5): c.604G>A, p.(Asp202Asn). The allele frequency (AF) for the variant in the 100kGP data set is 0.0115% (18/156,390).

## Discussion

We report here the first Brazilian patients (patient I, patient II) with SCAR32 due to bi‐allelic *PRDX3* mutations, replicating the pathogenic status of a known variant in the homozygous state as well as identifying novel pathogenic variants. SCAR32 represents a rare form of SCA, until now, only described in India, Morocco and Europe, particularly in Turkey (Table 1). All presented cases from the Brazilian and Indian populations expand the phenotypic spectrum of the disease by displaying prominent neuroradiological findings and hormonal dysfunction with the need for chorionic gonadotropin (HCG) replacement therapy. The Indian patient interestingly is the fourth youngest individual to date presenting with SCAR32 before 2 years of age, confirming that it can present at a postnatal age of onset. This observation further suggests that SCAR32, although rare, should be included in the differential diagnosis of sporadic or recessive childhood and adolescent‐onset pure and complex cerebellar ataxia.

The homozygous missense variant identified in our case, p.Asp202Asn, has been previously described in two unrelated families of Kurdish origin. Its identification in a Brazilian family suggests that this is not a population‐specific variant and may in fact be more widespread than originally thought especially in Latino and UK populations as the allele frequency of the variant is highest in these populations, according to our inspection of different variant frequency databases (Table S1).^1^ Additionally, the resulting variant (C>T) is at a cytosine‐guanine (CpG) dinucleotide, long known to be a hotspot for pathological mutation in the human genome, possibly explaining its recurrence due to deamination of methylated‐C type mechanism. Therefore, we estimate that there would be many more patients yet to be diagnosed with ataxia due to p.Asp202Asn mutation. The mutation is located in the PRX_Tyrp2cys domain of the protein at a region of high conservation in the dimer interface located between two PRDX3 subunits of the dodecamer ring and causes a complete absence of the protein.^1^ The extreme variance in age of onset, ranging from birth to 35 years in only a handful of cases, which seems to characterize both nonsense and missense mutations in *PRDX3*, is noteworthy and remains to be adequately explained at a pathophysiological level. All PRDX3 variants reported to date act via loss‐of‐function, as the mutant proteins are absent in patients' fibroblasts, leading to complete loss of the enzyme. It is expected that in the case of family 2, with two compound heterozygous variants, the p.Leu8Cysfs*10 is a loss‐of‐function variant depleting the protein, however, it is possible that is compensated to some extent by the p.Gly180Ser variant, hence delaying the age of onset of disease. Previously, in patient fibroblasts, the absence of PRDX3 was accompanied by reductions in PRDX5, a paralogue of PRDX3, and glutathione peroxidase activity, suggesting a coregulatory connection between PRDX3 and PRDX5, as well as glutathione peroxidase.^1^ This would explain why in some patients a compensatory effect predominates instead, mitigating the effects of PRDX3 deficiency and possibly delaying the age of onset of the disease.

Interestingly, patients with variants identified in the later part of the thioredoxin domain of the PRDX3 protein present with an early onset of the disease. Even if no clear genotype–phenotype correlations exist to date (Figure S1), it is possible that variants positioned within the CXXC motifs, that serve as catalytic active sites for the enzyme activity, result in a younger age of onset of the disease.

The presence of cerebellar atrophy is a ubiquitous feature in patients with bi‐allelic *PRDX3* mutations.^1^ One of the original simplex cases also had significant brainstem atrophy. Moreover, bilateral symmetric T2‐hyperintensity at the level of the medullary olives was noted in two cases.^1^ Subsequent reports brought attention to the presence of cerebellar cortical hyperintensity, T2‐hyperintense formation reticularis and dentate nucleus, T1‐hyperintense substantia nigra as well as transversal stripes of the pons.^4^, ^6^ The present report further highlights the extent of brainstem degeneration in PRDX3 patients.^9^, ^10^

In conclusion, our study reports three novel patients with rare PRDX3‐related SCAR, mainly in the Brazilian ataxia population, and hence expands the phenotypic spectrum of the disease, demonstrating that it can present with features suggestive of cerebellar cortical hyperintensities that link to early onset neurodegeneration.

## Conflict of interest

G.K. has received research grants from Genesis Pharma and Teva, consultation fees, advisory boards and honoraria from Genzyme, Genesis Pharma, Teva and Novartis. All other authors report no disclosures relevant to the manuscript.

## Supporting information


**Figure S1.** Genotype–phenotype correlations in recessive spinocerebellar ataxia type 32 (SCAR32). (A) Age of onset in an SCAR32 cohort. (B) variant type (missense, frameshift, non‐sense) carried in an SCAR32 cohort and (C) correlation between Scale for assessment and Rating of Ataxia (SARA) score and disease duration.

**Table S1.** In silico predictions and allele frequency of the identified PRDX3 variants in publicly available population databases and ACMG criteria classification.

## Data Availability

The authors confirm that the data supporting the findings of this study are available within the article.

## References

[1] Rebelo AP , Eidhof I , Cintra VP , et al. Biallelic loss‐of‐function variations in PRDX3 cause cerebellar ataxia. Brain. 2021;144(5):1467‐1481. 33889951 10.1093/brain/awab071

[2] Huh JY , Kim Y , Jeong J , et al. Peroxiredoxin 3 is a key molecule regulating adipocyte oxidative stress, mitochondrial biogenesis, and adipokine expression. Antioxid Redox Signal. 2012;16(3):229‐243. 21902452 10.1089/ars.2010.3766 PMC3234662

[3] Li L , Shoji W , Takano H , et al. Increased susceptibility of MER5 (peroxiredoxin III) knockout mice to LPS‐induced oxidative stress. Biochem Biophys Res Commun. 2007;355(3):715‐721. 17316558 10.1016/j.bbrc.2007.02.022

[4] Martinez‐Rubio D , Hinarejos I , Sancho P , et al. Mutations, genes, and phenotypes related to movement disorders and ataxias. Int J Mol Sci. 2022;23(19):11847. 36233161 10.3390/ijms231911847 PMC9570320

[5] Martinez‐Rubio D , Rodriguez‐Prieto A , Sancho P , et al. Protein misfolding and clearance in the pathogenesis of a new infantile onset ataxia caused by mutations in PRDX3. Hum Mol Genet. 2022;31(22):3897‐3913. 35766882 10.1093/hmg/ddac146 PMC9652108

[6] Rebelo AP , Bender B , Haack TB , et al. Expanding PRDX3 disease: broad range of onset age and infratentorial MRI signal changes. Brain. 2022;145(10):e95‐e98. 35792670 10.1093/brain/awac240 PMC10233235

[7] Rafeeq MM , Umair M , Bilal M , et al. A novel biallelic variant further delineates PRDX3‐related autosomal recessive cerebellar ataxia. Neurogenetics. 2023;24(1):55‐60. 36190665 10.1007/s10048-022-00701-9

[8] Efthymiou S , Salpietro V , Malintan N , et al. Biallelic mutations in neurofascin cause neurodevelopmental impairment and peripheral demyelination. Brain. 2019;142(10):2948‐2964. 31501903 10.1093/brain/awz248 PMC6763744

[9] Matsusue E , Fujii S , Kanasaki Y , Kaminou T , Ohama E , Ogawa T . Cerebellar lesions in multiple system atrophy: postmortem MR imaging‐pathologic correlations. AJNR Am J Neuroradiol. 2009;30(9):1725‐1730. 19541777 10.3174/ajnr.A1662 PMC7051491

[10] Higashi M , Ozaki K , Hattori T , et al. A diagnostic decision tree for adult cerebellar ataxia based on pontine magnetic resonance imaging. J Neurol Sci. 2018;387:187‐195. 29571861 10.1016/j.jns.2018.02.022

