# Supplementary figures and images for: Pure cerebellar ataxia due to bi‐allelic PRDX3 variants including recurring p.Asp202Asn
Source: Ann Clin Transl Neurol. 2023 Aug 8;10(10):1910–6. doi: 10.1002/acn3.51874 (PMC10578881; doi:10.1002/acn3.51874)

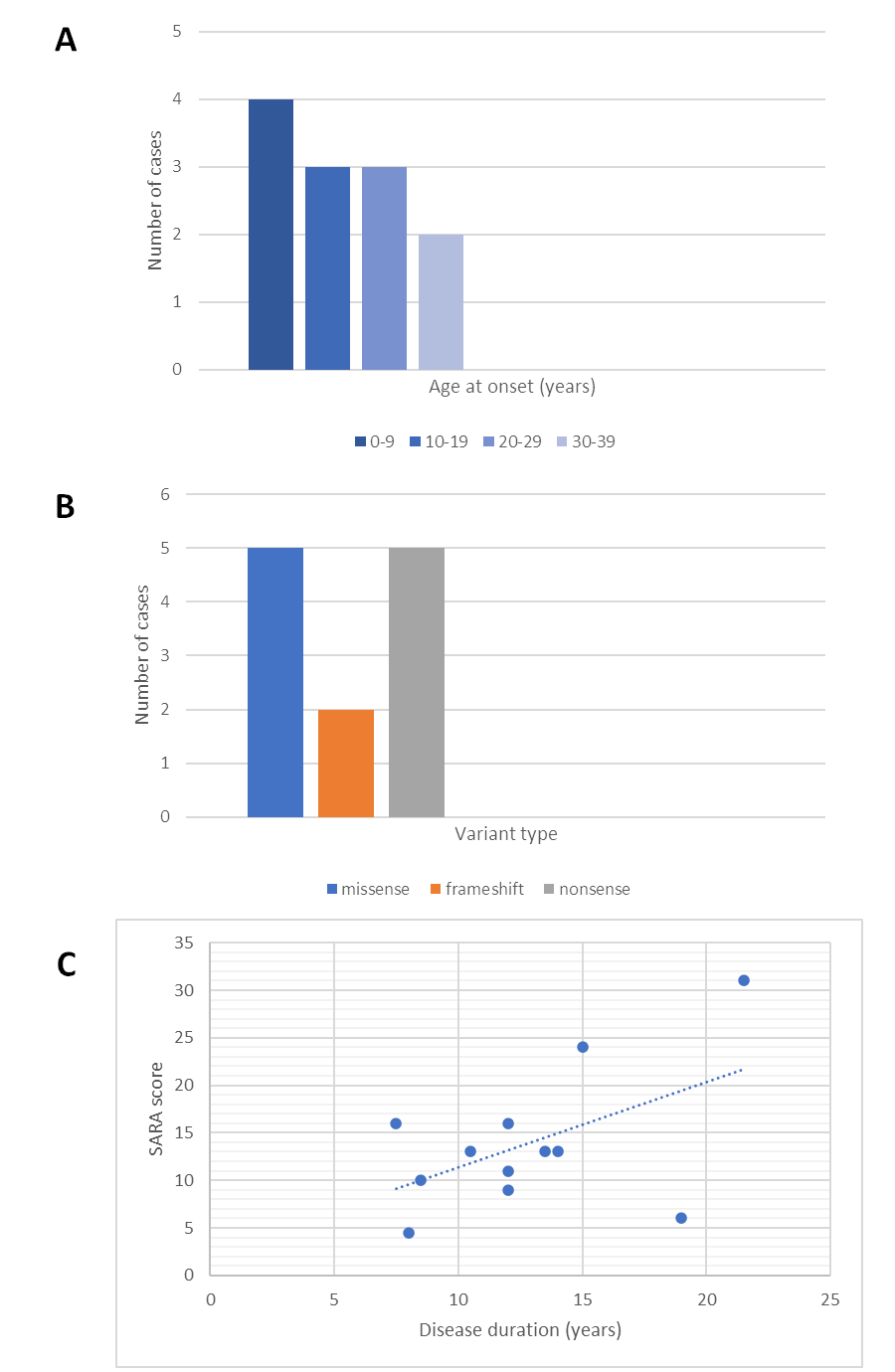

Supplement: Supplementary file 1 — Figure S1. Genotype–phenotype correlations in recessive spinocerebellar ataxia type 32 (SCAR32). (A) Age of onset in an SCAR32 cohort. (B) variant type (missense, frameshift, non‐sense) carried in an SCAR32 cohort and (C) correlation between Scale for assessment and Rating of Ataxia (SARA) score and disease duration. [file ACN3-10-1910-s002.tif]
